# How age and linguistic competence alter the interplay of perceptual and cognitive factors when listening to conversations in a noisy environment

**DOI:** 10.3389/fnsys.2014.00021

**Published:** 2014-02-25

**Authors:** Meital Avivi-Reich, Meredyth Daneman, Bruce A. Schneider

**Affiliations:** Human Communication Laboratory, Department of Psychology, University of Toronto MississaugaMississauga, ON, Canada

**Keywords:** age, nonnative listeners, speech comprehension, spatial separation, hearing, multitalker discourse, auditory-cognitive interaction, hearing loss

## Abstract

Multi-talker conversations challenge the perceptual and cognitive capabilities of older adults and those listening in their second language (L2). In older adults these difficulties could reflect declines in the auditory, cognitive, or linguistic processes supporting speech comprehension. The tendency of L2 listeners to invoke some of the semantic and syntactic processes from their first language (L1) may interfere with speech comprehension in L2. These challenges might also force them to reorganize the ways in which they perceive and process speech, thereby altering the balance between the contributions of bottom-up vs. top-down processes to speech comprehension. Younger and older L1s as well as young L2s listened to conversations played against a babble background, with or without spatial separation between the talkers and masker, when the spatial positions of the stimuli were specified either by loudspeaker placements (real location), or through use of the precedence effect (virtual location). After listening to a conversation, the participants were asked to answer questions regarding its content. Individual hearing differences were compensated for by creating the same degree of difficulty in identifying individual words in babble. Once compensation was applied, the number of questions correctly answered increased when a real or virtual spatial separation was introduced between babble and talkers. There was no evidence that performance differed between real and virtual locations. The contribution of vocabulary knowledge to dialog comprehension was found to be larger in the virtual conditions than in the real whereas the contribution of reading comprehension skill did not depend on the listening environment but rather differed as a function of age and language proficiency. The results indicate that the acoustic scene and the cognitive and linguistic competencies of listeners modulate how and when top-down resources are engaged in aid of speech comprehension.

## Introduction

Conversations with friends, co-workers, healthcare providers, and others often occur in noisy environments (e.g., malls, restaurants, stores, offices) in which there are a number of different sound sources that could interfere with one's ability to communicate effectively. In particular, the presence of other talkers, who are not part of the conversation, can be particularly distracting when one is trying to follow a conversation between two or more people. Such multi-talker auditory scenes increase the complexity of both the perceptual and cognitive processes required for comprehension. To effectively follow a multi-talker conversation, the listener needs to perceptually segregate the talkers from one another, efficiently switch attention from one talker to another, keep track of what was said by whom, extract the meaning of each utterance, store this information in memory for future use, integrate incoming information with what each conversational participant has said or done in the past, and draw on the listener's own knowledge of the conversation's topic to extract general themes and ideas (Murphy et al., 2006; Schneider et al., 2010). In other words, fully comprehending what is going on in a conversation requires the smooth and rapid coordination of a number of auditory and cognitive processes. Hence, it is not surprising that people in general find such situations stressful, and that older individuals, whose auditory and cognitive systems may be in decline, and even young, healthy listeners who are operating in their second or third language, find such situations particularly devastating.

To experimentally determine the reasons why people may find it difficult to follow conversations in noisy situations, we need laboratory simulations of ecologically-relevant listening environments and tasks, where we can control and manipulate relevant variables such as the nature and number of competing sound sources, their spatial locations, and the signal-to-noise ratios (SNRs) under which they are presented. By far, most of the studies designed to evaluate the relative contribution of perceptual and cognitive factors in hearing involve simple word or sentence recognition (see review by Humes et al., 2012). In such studies the target stimuli are either words or sentences and the listeners are simply asked to repeat them (e.g., George et al., 2007; Francis, 2010). The ability to repeat target words is taken as indicating speech is understood (Humes and Dubno, 2010). Comprehending what is happening in a conversation, as we have argued above, requires much more than simply being able to repeat the words being spoken. Hence, there is a substantial difference between speech recognition and speech comprehension (Schneider, 2011). A review of studies taking a correlational approach to the relationships among perception, cognition and speech recognition suggests that individual differences in speech recognition cannot be explained fully by the auditory or the cognitive factors that have been considered (age, pure tone thresholds, spectral and temporal processing, intensity coding, and cognitive processing; see Houtgast and Festen, 2008 for a review). Such studies, while they provide important information that may shed light on some of the processes needed for speech recognition (e.g., stream segregation, morpheme identification, lexical access), are limited with respect to their ability to address the role played by the higher-order cognitive processes required to successfully comprehend a conversation (e.g., attention switching, information integration, memory).

Although following a conversation in complex auditory scenes is a challenging task for all listeners, this task seems to be disproportionally harder for older adults (Murphy et al., 2006), and most likely for people listening in their second language (L2). Difficulties experienced by older adults could reflect age-related declines in the auditory, cognitive, and/or linguistic processes supporting spoken language comprehension. Age related changes occurring at different levels of the auditory system (e.g., elevated hearing thresholds, loss of neural synchrony, see Schneider, 1997; Schneider and Pichora-Fuller, 2000 for reviews), may also be accompanied by age-related changes in the cognitive processes related to speech comprehension. The cognitive aging literature notes that there are age-related reductions in the ability to focus attention and inhibit irrelevant sources (Hasher and Zacks, 1988; for a review, see Schneider et al., 2007), as well as evidence suggesting a general slowing of cognitive processing (e.g., Salthouse, 1996). Recent studies show that stream segregation may take a longer time to emerge in older adults than in younger adults in the presence of speech and speech-like maskers (Ben-David et al., 2012). In addition, although linguistic knowledge has been found to be relatively preserved in older age (Burke and Shafto, 2008), it is possible that under stressful and difficult listening situations, older adults may experience reduced capability to utilize their linguistic knowledge and skills in order to enhance speech comprehension because of age-related declines in executive functions (Clarys et al., 2009). Such age-related changes in auditory and cognitive processes may require a reorganization of the way information is processed in the brain. A number of studies have shown that older adults often engage different neural circuitry than that employed by younger adults when performing a task (Harris et al., 2009; Wong et al., 2009). Hence it is likely the relative contribution of different auditory and cognitive processes will be affected by aging (Schneider et al., 2010; Wingfield and Tun, 2007). One of the objectives of the current research is to determine how age, linguistic status, and the nature of the auditory scene differentially engage the perceptual and cognitive processes that contribute to speech comprehension.

A couple of relatively recent studies examined age-related changes in speech comprehension using longer and more complex tasks such as monologs and dialogs in order to gain a fuller understanding of the contribution of both the cognitive and perceptual processes involved in speech comprehension, and possible interactions among them. Schneider et al. (2000) asked both younger and older adults to listen to monologs and answer multiple-choice questions regarding their content at the end of each one. The results showed that age-related declines in the ability to process and remember a monolog could be eliminated when individual adjustments are made to compensate for speech recognition thresholds in younger and older listeners. Murphy et al. (2006) adapted Schneider et al.'s methodology to study the ability of younger and older adults to follow two-talker conversations instead of single-talker monologs. They selected a series of engaging one-act plays, each involving dialog between two characters of the same gender. Participants listened to the dialogs either in quiet or in a background of multi-talker babble noise. After listening to a 10–15 min dialog, participants answered a set of 10 multiple-choice questions that tested their comprehension and/or memory of details about the conversation. In Experiments 1–3, the talkers were separated by 9° or 45° azimuth in order to simulate a typical conversation between two talkers who necessarily have different spatial locations. However, in a control experiment, no such spatial separation was present (equivalent to a radio play). Their results indicated that older adults were able to answer fewer questions than younger adults in this listening-to-conversation task when both age groups were tested under identical stimulus conditions (Experiment 1) and that this age difference could be reduced but not eliminated when the listening situation is adjusted to make it equally difficult for younger and older adults to hear individual words when the two talkers are spatially separated (Experiments 2 and 3). The results of their last experiment (Experiment 4) showed that the age effect could be eliminated when listening situations were individually adjusted and there was no spatial separation present between talkers.

These results provided some evidence that older adults are indeed less skilled than younger adults at extracting and remembering information from a two-person conversation if adjustments have not been made for their poorer speech recognition thresholds. In addition, the consistent age difference, which was found as long as spatial separation between the two talkers was present, even after compensations had been made for the older listeners' deficits in hearing individual words, suggests that older adults might not be able to benefit as much from the full range of acoustic cues available with real spatial separation. A number of studies have shown that the improvement in speech reception thresholds that occurs when targets and maskers are spatially-separated rather than co-located, is significantly larger for young normal-hearing adults than for older, hearing-impaired adults (Neher et al., 2011). This inability of older and/or hearing-impaired adults to benefit from spatial separation could make it more difficult for them to establish and maintain stream segregation. In turn, increased stream segregation difficulties are likely to reduce the fidelity of the bottom-up, acoustic information, thereby requiring a greater degree of attentional investment at the perceptual level (Neher et al., 2009). As a result a change in the balance between the contributions of bottom-up vs. top-down processes may occur to compensate for the loss of fidelity in the neural representation of the acoustic signal. It is reasonable to assume that as a result of the changes mentioned, more weight will be given to top-down processes as the use of bottom-up information becomes limited. In particular, when there is interference with the bottom-up, stimulus-driven processes leading to lexical access, the listener may come to depend more on those top-down processes, such as vocabulary knowledge, that could be used to aid lexical access. Hence we might expect to find the correlation between vocabulary knowledge and speech comprehension to increase as the auditory scene becomes more complex. In addition, the deployment of attentional resources to aid lexical access could make it more difficult for listeners to engage higher-order modality-independent processes (such as those involved in reading comprehension) to help them to understand and retain what they have heard. However, to our knowledge, there have been no attempts to investigate the degree to which listening difficulties alter the relative balance between bottom-up and top-down processing in ecologically-valid listening situations, nor have there been attempts to determine the degree to which the contribution of different levels of processing to speech comprehension in such situations are modulated by the characteristics of the auditory scene, and the age and linguistic competence of the listener.

In order to further explore the sources of the age difference in the ability to comprehend and recall a dialog when two talkers are spatially separated, we used the precedence effect to change the virtual location of each talker. A number of studies have shown that if the same sound is played over two loudspeakers located to the left and right of the listener, with the sound on the left loudspeaker lagging behind that on the right, the listener perceives the sound as emanating from the right and vice versa (e.g., Rakerd et al., 2006). Because the sound is played over both loudspeakers, a virtual spatial separation is achieved without altering the SNR at each ear. Hence a perceived spatial separation can be achieved even though there is a substantial reduction in the auditory cues supporting spatial separation (e.g., no head shadow effect). Moreover, Cranford et al. (1990) have shown that when the precedence effect is used to specify the virtual locations of sounds, both younger and older adults experienced the sound as emanating from the side where the leading loudspeaker was positioned The precedence effect has been used in a number of studies related to informational masking as a way of achieving a perceived (virtual) separation between sound sources without substantially affecting the SNR at each ear of the listener (e.g., Freyman et al., 1999; Li et al., 2004) and it has been shown that both younger and older adults reap the same degree of benefit from perceived separation (Li et al., 2004). However, using the precedence effect to achieve and maintain a virtual spatial separation among sound sources may require that a larger proportion of attentional resources be allocated to stream segregation since the sound sources under the precedence effect are perceived as more diffuse and cannot be precisely located in space. This, in turn, may alter the balance between the top-down and bottom-up processes involved in speech recognition.

Nonnative listeners also constitute a group that experiences considerable difficulty when attempting to follow a conversation in their second language (L2) in the presence of background noise. Nonnative young listeners are not likely to differ from native young listeners using their first language (L1) with respect to basic auditory and cognitive abilities. However, nonnative listeners of a language tend to have lower scores than native listeners on a number of speech-perception measures (Mayo et al., 1997; Bradlow and Pisoni, 1999; Meador et al., 2000; Bradlow and Bent, 2002; Cooke et al., 2008; Rogers and Lopez, 2008; Ezzatian et al., 2010). This difference in performance is influenced by several factors, such as duration of exposure to L2, degree of similarity between L1 and L2, knowledge of the L2 vocabulary and grammatical structure, frequency and extent of L2 use, etc. The acoustic–phonetic characteristics of a second language, which was acquired or learned at a later age than their L1, may not be fully acquired (e.g., Florentine, 1985; Mayo et al., 1997), resulting in a reduced ability to discriminate fine phonemic information, such as phonetic contrasts and phonemic categories which are crucial for successful speech perception (Bradlow and Pisoni, 1999; Meador et al., 2000). In regard to listening in noise, previous literature on young nonnative listeners suggests that nonnative listeners are less able to make use of a language mismatch between masking and target stimuli to facilitate masking release (Brouwer et al., 2012). In the case of nonnative listeners, it is reasonable to assume that the difficulties they experience in L2 environments may be due to the fact their L2 semantic and linguistic processes may not be completely differentiated from the semantic and syntactic processes that are usually invoked when listening in their L1 (Kroll and Steward, 1994).

In the current study we choose to further explore the effect of age and linguistic status on the ability to successfully follow a two-talker conversation (dialog) conducted in a babble background noise with the locations of the sound sources being either virtual or real, and with the two talkers being spatially separated or co-located. Native-English younger and older listeners and young nonnative-English listeners were asked to listen to conversations played against a babble background noise and to answer questions regarding their content. Individual hearing differences were compensated for by creating the same degree of difficulty in identifying individual words in a babble background when there was little or no contextual support for word recognition. Two measures of individual differences in linguistic competence were included; a measure of vocabulary knowledge (the Mill Hill; Raven, 1965) and a measure of reading comprehension skill (the Nelson-Denny; Brown et al., 1981)^1^. If listening difficulty, linguistic status, or age alters the relative contribution of bottom-up and top-down processes, and differentially affects the various stages in the speech processing network, we might expect that the degree to which these two factors are correlated with the ability to comprehend and remember dialogs to vary across groups and listening conditions.

## Materials and methods

### Participants

The participants were 24 normal hearing younger adults who are native-English listeners (mean age: 21.26 years; *SD*: 3.02), 24 normal hearing older adults who are native–English listeners (mean age: 69.7 years; *SD*: 4.6), and 24 normal hearing young adults who are nonnative-English listeners (mean age: 21.04 years; *SD*: 1.71). Native-English listeners were all born and raised in a country in which the primary language was English and were not fluent in any other language at the time of participation. Nonnative-English listeners were those who first became immersed in an English speaking environment after the age of 14. One older listener, who found the noisy background in the study to be uncomfortable, withdrew from the experiment and had to be replaced. The young participants were volunteers recruited from the students and staff at the University of Toronto Mississauga. The older participants were volunteers from the local community. All participants were asked to complete a questionnaire regarding their general health, hearing, vision, and cognitive status. Only participants who reported that they were in good health and had no history of serious pathology (e.g., stroke, head injury, neurological disease, seizures, and the like) were included. All participants reported having a normal or corrected vision and were asked to use their correcting lenses when necessary. None of the participants had any history of hearing disorders, and none used hearing aids. The studies reported here were approved by the Ethics Review Board of the University of Toronto.

During each participant's first session at the lab we administrated audiometric thresholds, the Nelson-Denny reading comprehension test (Brown et al., 1981) and the Mill Hill test of vocabulary knowledge (Raven, 1965). The dialogs, along with the babble thresholds and the low-context R-SPIN thresholds were administered over the next two experimental sessions (three dialogs per session). Tests were administered in a double-walled sound-attenuating chamber. All participants were paid $10/h for their participation.

### Hearing measures

#### Audiometric testing

**Figure 1:**
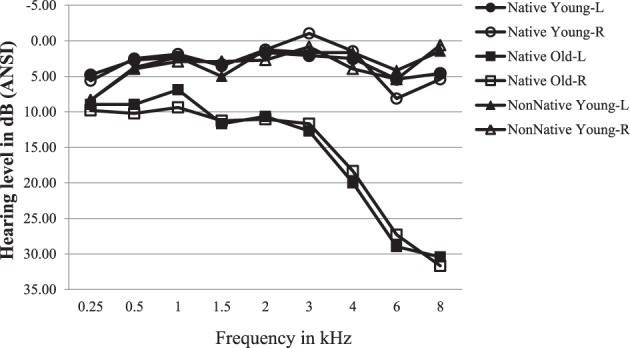
**Average audiograms for the three groups of participants are shown for the right and the left ears**. ANSI, American National Standards Institute.

Pure-tone air-conduction thresholds were measured at nine frequencies (0.25–8 kHz) for both ears using an Interacoustics Model AC5 audiometer (Interacoustic, Assens, Denmark). All younger participants were required to have pure tone air-conduction thresholds 15 dB HL or lower, between 0.25 and 8 kHz in both ears. Young participants with a threshold of 20 dB HL at a single frequency were not excluded from the study. Older participants were required to have a pure tone thresholds 25 dB HL or lower from 0.25 to 3 kHz and 35 dB HL or lower for frequencies <6 kHz. Participants who demonstrated unbalanced hearing (more than a 15 dB difference between ears under one or more frequencies) were excluded from participation. Older adults with hearing in the range described are usually considered to have normal hearing for their age. However, it is acknowledged that older adults' hearing changes and deteriorates with age and is not equivalent to that of younger adults (Wingfield et al., 1985; Fitzgibbons and Gordon-Salant, 1996; Wingfield, 1996; Schneider and Pichora-Fuller, 2001; Glyde et al., 2011). The average audiograms for the three groups of participants are shown for the right and the left ears in Figure 1. The two groups of young adults had equivalent hearing levels at all frequencies. Hearing levels for older adults were about 7 dB poorer than those of the younger adults at frequencies ≤3 kHz, with the younger-older difference increasing as a function of frequency for frequencies >3 kHz.

#### Babble threshold test

**Figure 2:**
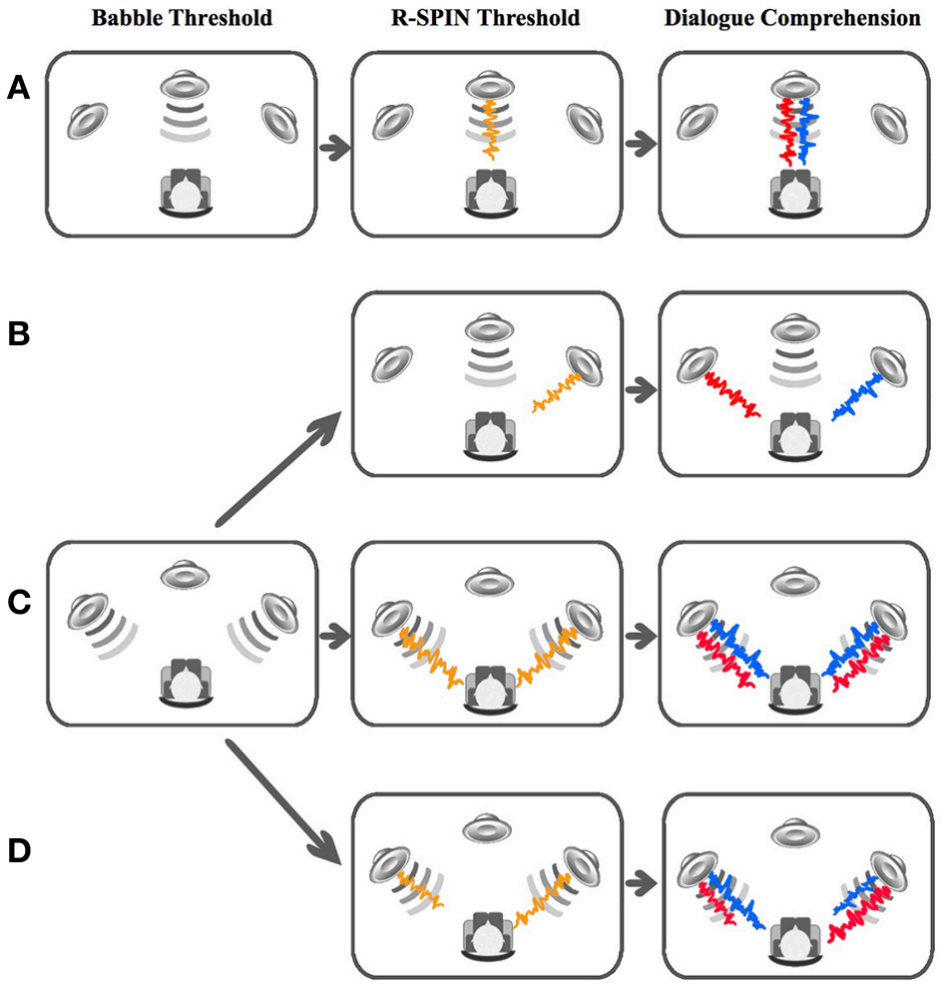
**The left column specifies the two babble thresholds collected, and under what conditions they were used to adjust the signal level; the middle column specifies the conditions under which R-SPIN thresholds were obtained; and the right column illustrates the four dialog comprehension test conditions in the experiment.** The top row **(A)** specifies the babble, R-SPIN and dialog comprehension scenarios for the real no-separation condition. The three other conditions share the same babble threshold but differ from each other in respect to the R-SPIN and the dialog comprehension tasks. The second row from the top **(B)** illustrates the R-SPIN and dialog comprehension for the real spatial separation condition, the third row **(C)** illustrates the settings for the virtual no-separation and the fourth **(D)** specifies the settings for the virtual spatial separation. Orange stands for R-SPIN, red stands for talker 1, blue for talker 2, and gray for babble.

The adaptive two-interval forced choice procedure employed by Schneider et al. (2000) was used to measure individual detection thresholds for the 12-talker babble masker used in this experiment. In this procedure, a 1.5 s babble segment was randomly presented in one of two intervals which were separated by a 1.5-s silent period. Two lights on the button box indicated the occurrence of each interval, and the listener's task was to identify the interval containing the babble segment by pressing the corresponding button. Immediate feedback was provided after each press. We used an adaptive two down one up procedure (Levitt, 1971) to determine the babble threshold corresponding to the 79% point on the psychometric function. Two different babble thresholds were determined for each individual. First, a babble threshold was determined when the babble was presented over a single central loudspeaker. The sound levels of the speech signals for the condition in which the voices of both talkers were presented only over the central loudspeaker (no separation, single loudspeaker condition) were individually set to be 45 dB above this babble threshold (sensation level, SL, of 45 dB). A second babble threshold was determined when the babble was played simultaneously over two loudspeakers located 45° to the right and left of the listeners. This babble threshold was used to adjust the intensity of speech signal to 45 dBSL for the conditions in which voices were presented over both lateral loudspeakers. For a graphic illustration of the two babble conditions see Figure 2, first column on the left.

#### R-SPIN test

In this test participants are asked to immediately repeat the last word of individual sentences presented to them in a multi-talker babble background. As in Schneider et al. (2000) and Murphy et al. (2006), we used the Revised Speech Perception in Noise (R-SPIN) test (Bilger et al., 1984) to individually determine the SNR producing 50% correct identification of final words of low-context sentences (e.g., Jane was thinking about the van.) presented in multitalker babble under both real and virtual location conditions. In the real no-separation condition, both the babble and R-SPIN sentences were played over the central loudspeaker. In the real separation conditions, the babble was presented over the central loudspeaker and the R-SPIN sentences were presented over the right loudspeaker. In the virtual no separation condition, both the babble and R-SPIN sentences were presented simultaneously over both loudspeakers. In the virtual separation conditions the babble was presented over both lateral loudspeakers simultaneously with the R-SPIN sentences also presented over both loudspeakers but with the sentences presented over the right loudspeaker leading the sentences presented over the left loudspeaker by 3 ms. Hence R-SPIN thresholds were determined for each of the conditions under which participants were tested. The R-SPIN thresholds estimated were rounded to units of 1 dB before being integrated into the calculation of the individually adjusted SNR which was used for presentation of the dialogs. If the estimated R-SPIN threshold was calculated to be exactly half way between two integer values rounding was conducted toward alleviating the SNR difficulty (e.g., 3.5 dB SNR was rounded to 4 dB SNR and −3.5 dB SNR to −3 dB SNR). For a graphic illustration of the four R-SPIN conditions see Figure 2, second column from the left.

#### Dialog comprehension task

Each participant was asked to listen to six dialogs presented in babble noise in a sound-attenuating chamber. These dialogs were created and previously used by Murphy et al. (2006). Each dialog was based on a published one-act play and had only two characters; in three of the six dialogs, the two characters were female, and in the other three, the two characters were male. At the end of each dialog, which was 10–15 min long, the participant was presented with a series of 10 multiple-choice questions regarding the contents of that dialog (for more information regarding the dialogs and the questions see Murphy et al., 2006). There were four conditions in the experiment: (a) Real spatial separation with the babble presented over the central loudspeaker, with the voice of one of the talkers presented over the left loudspeaker, and the voice of the other talker presented over the right loudspeaker; (b) Real, no spatial separation, with the babble, and the voices of the two talkers presented over the central loudspeaker only; (c) Virtual no spatial separation with the babble and two voices being played simultaneously over both lateral loudspeakers; (d) Virtual spatial separation with the babble presented simultaneously over both lateral loudspeakers, and the two voices also presented over both lateral loudspeakers with the perceived location of the two talkers manipulated using the precedence effect (the voice of talker one over the left loudspeaker playing 3 ms in advance over the same voice playing over the right loudspeaker, with the opposite timing arrangement for the second voice). For a graphic illustration of the four dialog comprehension conditions, see Figure 2 rightmost column. Half of the participants were tested in conditions a and c (separation conditions), the other half in conditions b and d (no separation conditions). Hence in this design, virtual vs. real location was a within-subject factor and spatial location was a between-subjects factor. The participants in each group were randomly assigned to one of the two spatial location conditions. The dialogs were presented in babble at an SNR which was individually adjusted per participant and condition based on his or her babble threshold and R-SPIN results according to the following calculations:

Dialogs were presented at babble threshold + 45 dB; Babble was presented at babble threshold + 45 dB − R-SPIN threshold + 21 dB.

At the end of each dialog, participants were asked to answer a set of 10 multiple-choice questions with four alternatives that were also constructed and previously used by Murphy et al. (2006). Each question referred to a specific item of information that was mentioned explicitly only once during the dialog.

### Language proficiency measures

#### Vocabulary knowledge

Participants were asked to complete the Mill Hill vocabulary test (Raven, 1965), which is a 20-item synonym test. In this test participants were required to match each test item with its closest synonym from six listed alternatives. No time restrains were applied. The extent of a person's vocabulary represents knowledge that can be used to facilitate word recognition. When listening becomes difficult, and the fidelity of the bottom-up information contributing to word identification becomes questionable, we might expect the role played by top-down knowledge (e.g., the extent of an individual's vocabulary) to increase. Hence we might expect the correlation between vocabulary knowledge and the number of dialog questions correctly answered to increase with listening difficulty.

#### Reading comprehension skill

The Nelson-Denny test (Brown et al., 1981) was used to assess the reading comprehension skills of each participant. In this test the participants had to read through a series of eight independent passages and answer multiple-choice questions regarding the content of the passages. This test includes a total of 36 questions and was limited to 20 min. Participants were instructed to complete as many questions as possible within the time given.

The six dialogs were administered over two sessions, three dialogs per session. Sessions were typically 1.5–2 h in duration and were completed within 2 weeks.

## Results

Table 1 presents the gender breakdown, mean age, educational level, Mill Hill test of vocabulary knowledge and Nelson-Denny test of reading comprehension results for each age group. One of the nonnative-English listeners had an R-SPIN threshold of 22 dB SNR in the virtual no separation condition. Since this value was more than three standard deviations above the mean for that group, this value was identified as an outlier and was replaced by the average R-SPIN threshold of the nonnative-English listeners group after excluding the outlier (6 dB SNR)^2^.

**Table 1:** **Demographic information (Mean Age and Years of Education, Gender Distribution, Mean Vocabulary, and Reading Comprehension Scores) and Mean Babble and R-SPIN thresholds for the participants in the two separation conditions divided into the three groups tested**.

	**Separation con**.							**No-separation con**.						
	**Younger natives**		**Older natives**		**Young nonnatives**		***p* < 0.05**	**Younger natives**		**Older natives**		**Young nonnatives**		***p* < 0.05**
	***M***	***SD***	***M***	***SD***	***M***	***SD***		***M***	***SD***	***M***	***SD***	***M***	***SD***	
Age	21.09	2.17	68.83	3.33	21.33	1.50	A, C	21.42	2.19	70.58	5.20	20.75	1.91	A, C
Education	15.58	2.27	15.85	2.44	14.67	1.50		16.17	2.29	14.58	2.39	14.25	1.48	B
Gender	10 F + 2 M		11 F + 1 M		9 F + 3 M			10 F + 2 M		7 F + 5 M		9 F + 3 M		
Vocabulary (max = 20)	13.25	2.34	15.00	1.35	8.58	2.11	A, B,C	14.33	1.23	15.25	2.22	9.17	3.64	B, C
Reading comprehension (max = 36)	26.33	7.13	19.92	3.90	18.08	5.73	A, B	24.42	4.87	22.92	7.59	15.83	7.27	B, C
Babble threshold from central loaudspeaker (dB SPL)								9.82	3.24	15.72	6.70	10.41	2.60	A, C
Babble threshold from lateral loaudspeakers (dB SPL)	5.23	1.47	11.68	4.02	6.40	3.08	A, C	6.19	3.13	11.91	7.48	6.27	2.92	A, C
R-SPIN threshold for real condition (dB)	−3.76	2.65	−1.52	2.29	−0.66	2.51	A, B	1.86	1.56	2.32	1.56	6.54	2.87	B, C
R-SPIN threshold for virtual condition (dB)	−1.40	1.62	1.30	1.40	4.13	1.72	A, B, C	1.36	0.92	1.92	1.47	7.29	4.72	B, C

*Statistically significant differences between groups are noted*.

*Max, maximum; dB, decibel; SPL, sound pressure level; R-SPIN, Revised Speech Perception in Noise*.

*A, A statistically significant difference between Younger Native-English and Older Native-English listeners*.

*B, A statistically significant difference between Younger Native-English and Young Nonnative-English listeners*.

*C, A statistically significant difference between Older Native-English and Young Nonnative-English listeners*.

### R-SPIN thresholds

**Figure 3:**
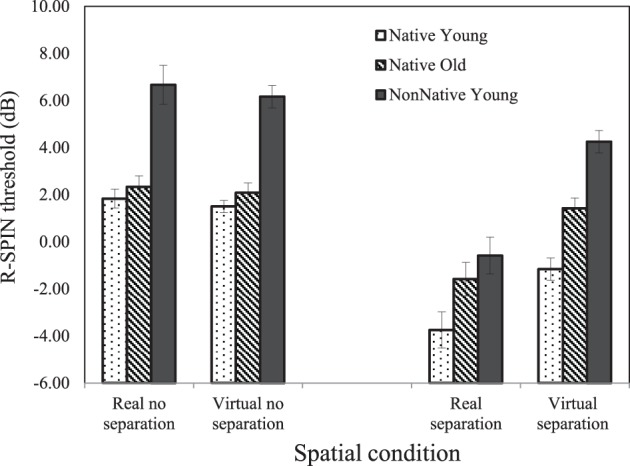
**Average R-SPIN thresholds (dB) calculated under each of the four spatial conditions**. Standard error bars are shown.

Figure 3 plots the average 50% correct R-SPIN thresholds (dB) as a function of separation condition and group for the native-English younger listeners (dotted rectangles), the native-English older listeners (lined rectangles), and the nonnative-English young listeners (solid rectangles). The SNR levels required for 50% correct repetition of the last word in low-context sentences was highest when there was no spatial separation vs. when there was a separation between the target sentences and the babble background, and were on average lower for real than for virtual locations. The R-SPIN thresholds also appear to be higher for young nonnative-English listeners than for older native-English listeners, who, in turn, had higher thresholds than the younger native listeners. In addition, the advantage due to spatial separation is larger when spatial location is real than when it is virtual. The benefit of separation over no separation on average was larger for the young nonnative-English listeners (5.18 dB) than both the younger (4.2 dB) and older natives-English listeners (2.18 dB).

A repeated measures ANOVA with two separation conditions (yes/no) and three groups (younger and older native-English listeners and young nonnative-English listeners) as between-subjects factors, and with the two types of spatial location (real/virtual) as a within-subject factor confirmed this description, showing a significant main effect of Separation Condition [*F*_(1, 66)_ = 97.613, *p* < 0.000], Group [*F*_(2, 66)_ = 51.539, *p* < 0.000] and Type of Location [real vs. virtual; *F*_(1, 66)_ = 30.451, *p* < 0.000] as well as a two-way Group by Separation interaction [*F*_(2, 66)_ = 3.559, *p* = 0.034] and a Type of Location by Separation interaction [*F*_(1, 66)_ = 46.367, *p* < 0.000]. No other effects were significant. *Post-hoc* tests with Sidak adjustment found that all three groups differed significantly from one another (*p* < 0.005 for all three pairwise comparisons).

**Figure 4:**
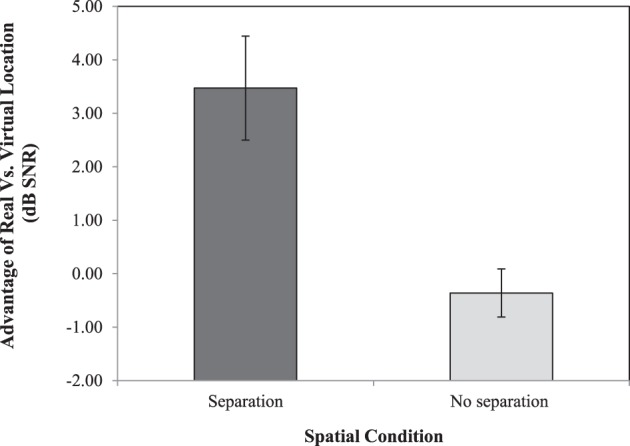
**Average advantage of real vs. virtual location cues (R-Spin threshold virtual—R-SPIN threshold real) for the two types of separation (target sentences and babble separated vs. co-located)**. Standard error bars are shown.

To better illustrate the nature of the Separation by Type of Location interaction, Figure 4 shows the advantage of real vs. virtual location cues (R-Spin threshold virtual—R-SPIN threshold real) for the two types of separation (target sentence and babble separated vs. co-located). This figure clearly indicates the advantage of real over virtual location is larger when the target sentence and babble were perceived to be spatially separated than when they were perceived to be co-located.

**Figure 5:**
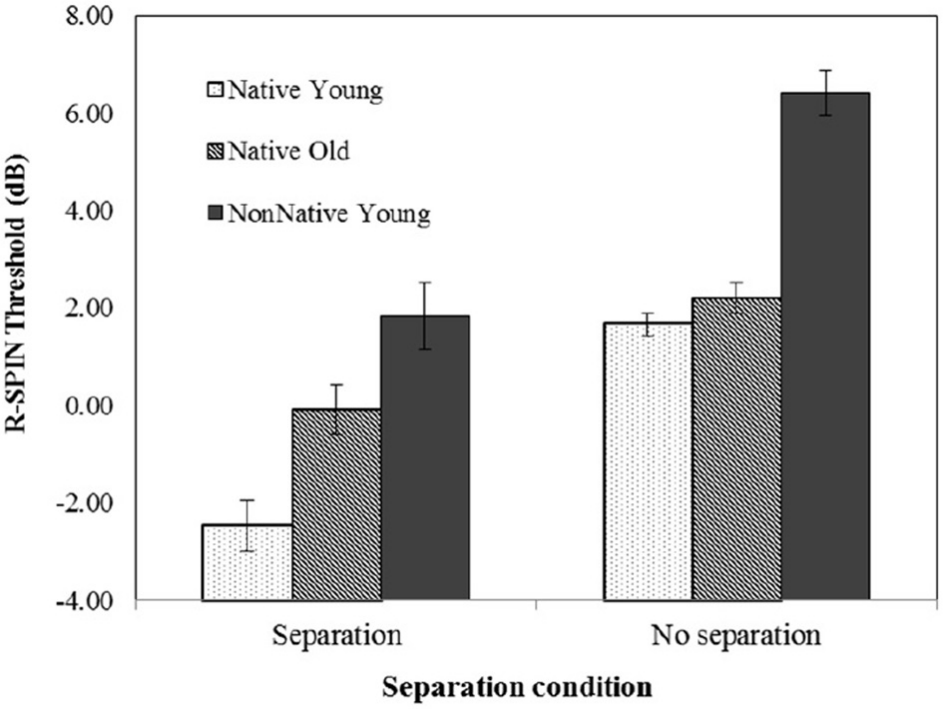
**Average R-SPIN thresholds (dB) calculated under each of the two spatial location conditions for each of the three groups**. Error bars are shown.

Figure 5 suggests that the Group by Separation interaction is due to the fact that the benefit due to spatial separation is smaller for older native listeners than either of the younger groups. An examination of the group by spatial separation interaction for older vs. younger native listeners found the benefit due to spatial separation to be significantly smaller for older natives than for younger natives [Group × Separation interaction: *F*_(1, 44)_ = 6.127, *p* = 0.017]. The Group × Separation interaction was also significant when the two groups were older natives and young nonnatives [*F*_(1, 44)_ = 4.927, *p* = 0.032], but not when young native listeners were compared to young nonnative listeners [*F*_(1, 44)_ < 1]. Figure 5 also suggests that when there is no separation, the R-SPIN thresholds are equivalent for both younger and older native listeners. A separate ANOVA on the no-separation condition revealed a significant effect of group [*F*_(2, 32)_ = 38.154, *p* < 0.001] with *post-hoc* tests with Sidak adjustment indicating that the nonnative English listeners differed significantly from both native groups (*p* < 0.001 in both cases) but that that the younger and older native English listeners did not differ significantly from one another. A comparable analysis of the data from the Separation Condition revealed a significant Group effect [*F*_(2, 33)_ = 20.842, *p* < 0.001] with the *post-hoc* tests with Sidak adjustment revealing that all three groups differed significantly from one another (*p* < 0.03 for all pairwise comparisons).

### Dialog comprehension results

**Figure 6:**
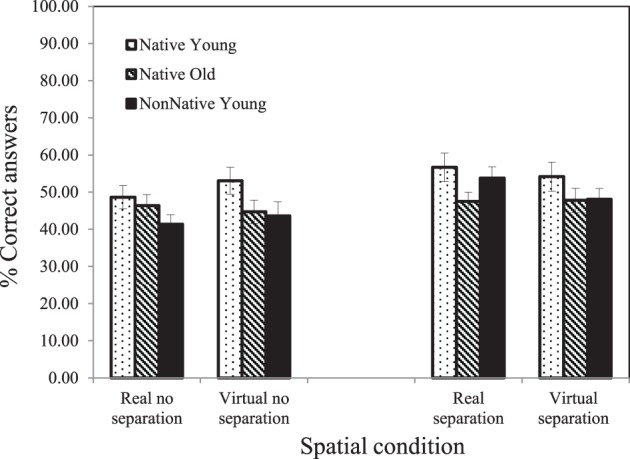
**Average percentage of correctly answered questions calculated under each of the four spatial conditions for each of the three groups**. Error bars are shown.

The main finding of interest was that once the SNR levels were adjusted based on the R-SPIN results, only small differences were found between the three groups tested in the amount of questions correctly answered (see Figure 6). In general the native-English young listeners seemed to perform slightly better than either the native-English older listeners or the nonnative-English young listeners. However, a 2-within-subject (real vs. virtual) × 2-between-subject (separation vs. no-separation) by 3-between-subject (younger and older native-English listeners, nonnative-English young listeners) ANOVA revealed only a significant main effect of Separation [*F*_(1, 66)_ = 4.671, *p* = 0.034] with performance being better when the voices were separated rather than co-located.

### The contribution of vocabulary knowledge and reading comprehension to dialog comprehension

**Figure 7:**
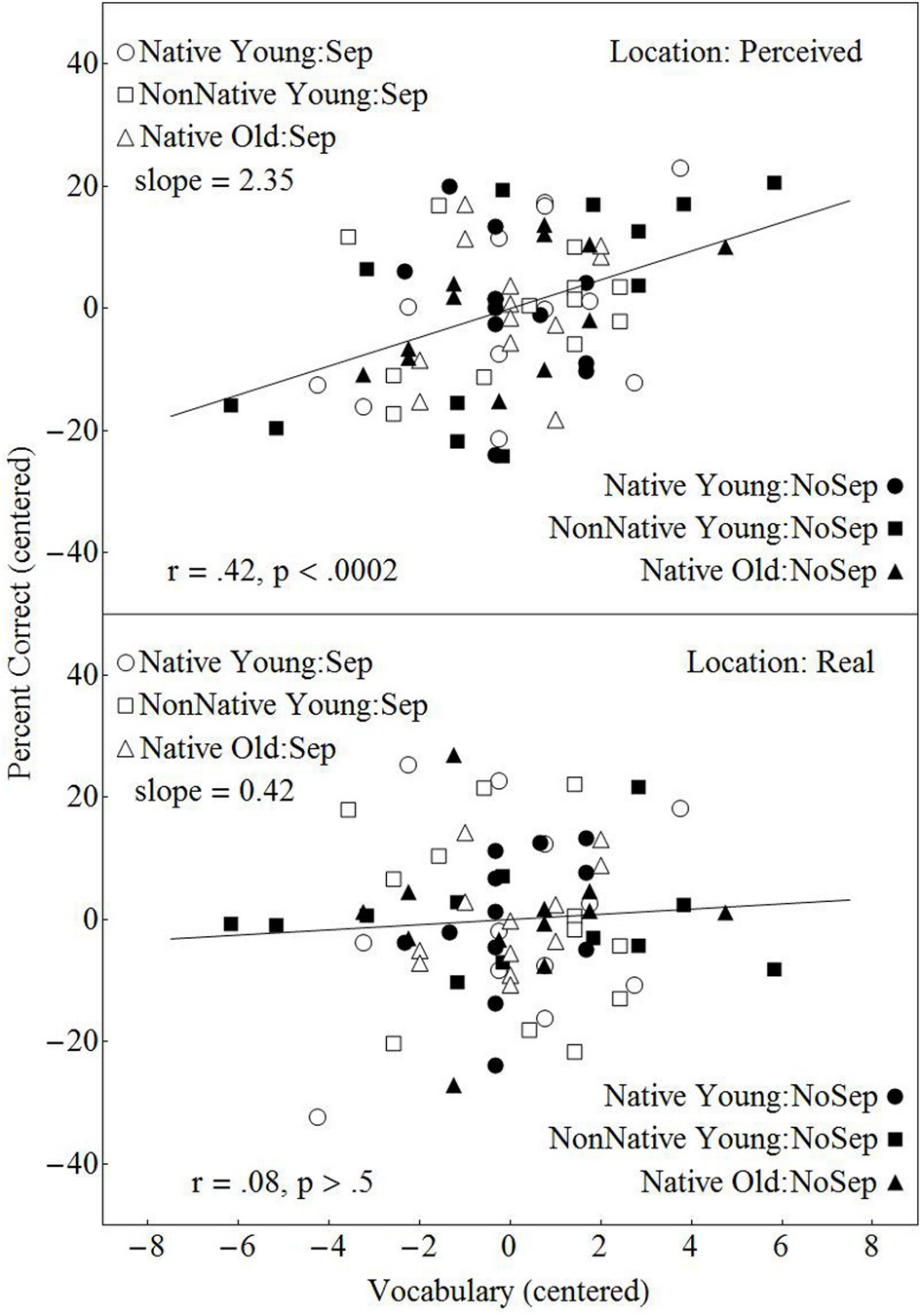
**Percentage of correctly answered dialog questions plotted against the individual performance on the Mill Hill vocabulary test after the contribution of reading comprehension to performance had been removed**. Both the adjusted number of questions answered and the Mill Hill scores are centered within each group. A least squares regressions line is presented for each of the two types of location.

We explored the degree to which the different levels of the factors (Separation, Group and Type of Location) were differentially associated with individual differences in vocabulary knowledge and in reading comprehension skill. First we examined whether individual differences in vocabulary knowledge were more predictive of the number of dialog comprehension questions answered correctly when the cues to spatial location were real, as opposed to virtual. Figure 7 relates the percentage of questions correctly answered as a function of vocabulary score separately for the virtual location conditions (upper panel), and the real location conditions (lower panel). In this figure, the vocabulary scores were first centered within each group of participants to normalize the vocabulary scores across the six groups of participants. Percent correct answers were also normalized within each of the 12 conditions in the experiment to eliminate the contribution of any residual effects of conditions on performance. Before conducting these regression analyses, we first removed any effect that individual differences in reading comprehension had on performance (see Appendix A). This allowed us to evaluate the effects of individual differences in vocabulary once the effects of reading comprehension on performance had been removed. Figure 7 shows that slope of the line relating percent correct to vocabulary knowledge is considerably higher for virtual location than real location. A regression analysis (see Appendix A) found vocabulary knowledge to be significantly related to dialog comprehension for the virtual conditions but not for the real location conditions, with the difference in correlation between the two (the interaction between vocabulary and Type of Location) also being significant [*F*_(1, 142)_ = 4.70, *p* = 0.03]. However, similar regression analyses failed to find any evidence that the relationship of vocabulary knowledge to percent correct differed between the no-separation conditions and the separation conditions, or among the three groups of participants (native-English younger listeners, native-English older listeners, and nonnative-English young listeners).

**Figure 8:**
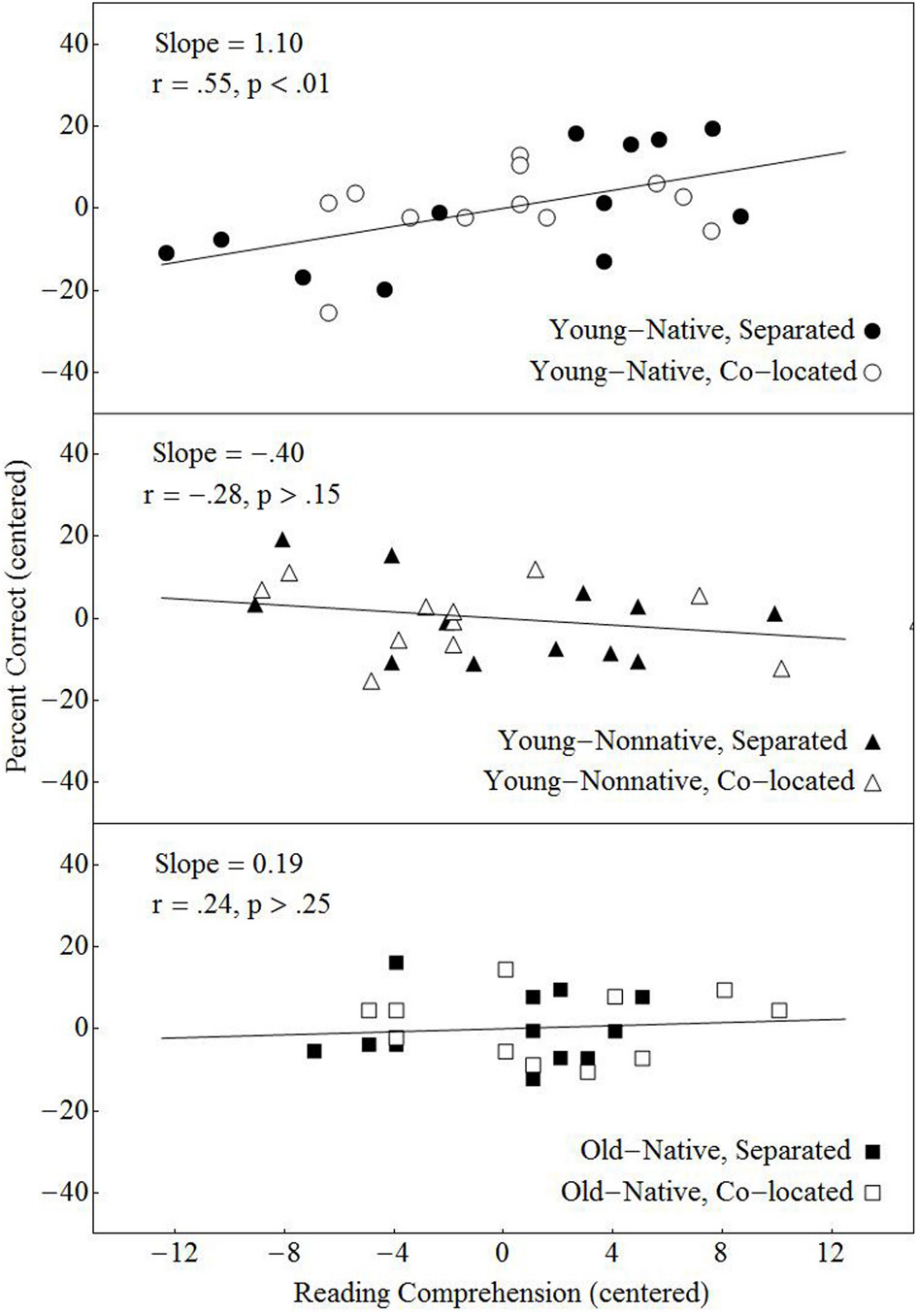
**Percentage of correctly answered dialog questions plotted against the individual performance on the Nelson-Denny reading comprehension test after the contribution of vocabulary to performance had been removed**. Both the adjusted number of questions answered and the Nelson Denny scores are centered within each group. A least squares regressions line is presented for each of the three groups.

A similar analysis was conducted to examine the contribution of reading comprehension skill to dialog comprehension. In particular, before evaluating the contribution of reading comprehension to performance, we first moved any effect that individual differences in vocabulary had on performance. The results of this analysis indicated that the contribution of reading comprehension skill to dialog comprehension did not differ between the virtual and real location conditions, nor did the contribution differ between the no-separation and separation conditions. However, the contribution of reading comprehension skill to performance on the dialog comprehension task did differ across the three groups. Figure 8 shows that reading comprehension was positively correlated with performance for the native-English younger listeners only. A regression analysis (see Appendix A) indicated that the strength of the relationship differed among the three groups, with Bonferroni—corrected pairwise comparisons confirming that the slopes differed significantly between young native-English and nonnative-English listeners.

We also examined whether either the vocabulary scores or the reading comprehension scores were related to R-SPIN in each of the 12 conditions (3 Groups × 2 Spatial Locations × 2 Types of Location). Similar analyses to those conducted for the percentage of correctly answered dialog questions failed to find any evidence that either vocabulary knowledge or reading comprehension skill could account for individual differences in the R-SPIN threshold values.

### Comparison of current data to previous data

Two of the conditions in the current study replicate two similar conditions which were found in the Murphy et al. (2006) study. In both the Murphy et al. and the current study, younger and older native-English listeners were volunteers recruited from the University Community and local neighborhood community, respectively. No nonnative-English listeners were tested in Murphy et al. The real separation condition in the present study is comparable to the high babble noise condition where the dialogs were presented from loudspeakers at 45° azimuth in Experiment 3 of Murphy et al. and the real no spatial separation condition replicates the condition in which the dialogs were presented once the spatial separation was removed in the last experiment reported (experiment 4) by Murphy et al. The R-SPIN thresholds and the dialog comprehension results of these two comparable conditions from both studies were analyzed separately using a Univariate Analysis of Variance with Age, Experiment and Separation as between-subjects variables. The results of this analysis revealed a significant main effect of Age [*F*_(7, 88)_ = 4.296, *p* = 0.041] but not of Experiment [*F*_(1, 88)_ = 0.963, *p* = 0.329] nor Separation [*F*_(1, 88)_ = 0.210, *p* = 0.648] on dialog comprehension performance. In addition there were no significant two- or three-way interactions among these three factors. Hence there is no evidence that the participants in this experiment differed in performance from those in Murphy et al.

A similar analysis which compared the R-SPIN thresholds calculated under the two comparable conditions in the two studies showed a significant effect of Age [*F*_(1, 88)_ = 125.94,*p* < 0.001] and Separation [*F*_(1, 88)_ = 504.45, *p* < 0.001]. As for the dialog comprehension performance, no significant effect of Experiment was found [*F*_(1, 88)_ = 0.089, *p* = 0.89], however a significant interaction was found between Age and Experiment [*F*_(1, 88)_ = 21.4, *p* = 0.039]. The latter interaction is probably a result of the younger adults in the current study performing slightly worse than the younger adults who participated in Murphy et al. and the older adults in the current study performing slightly better than the older adults who participated in Murphy et al. when there was no spatial separation. Overall, the evidence suggests that the current study successfully replicated the study conducted by Murphy et al. (2006) in regard to the two comparable conditions, and that participants in this study did not differ significantly from the participants in Murphy et al. with respect to their performance in both the dialog comprehension task and the R-SPIN word recognition task in those conditions which were comparable across the two experiments.

## Discussion

### Using the R-SPIN results as an index of speech recognition difficulties

The R-SPIN results indicate that R-SPIN thresholds are lower for real than for virtual location (see Figure 3). This is consistent with the hypothesis that it is more difficult to parse the auditory scene when the location of the sources is virtual rather than real. There is also evidence (see Figure 4) that the advantage of real over virtual location of stimuli is considerably larger in the presence of spatial separation. The R-SPIN results also indicate that nonnative-English young listeners and native-English older listeners find it more difficult to recognize words in babble than young native-English listeners (see Figure 3). Moreover the results show that older adults benefit less from spatial separation than native-English and nonnative-English younger listeners (see Figure 5).

The results also show that when the target sentence and babble appear to be co-located, younger and older native-English listeners have similar R-SPIN thresholds that are significantly lower than those of the nonnative-English listeners. However, when the target sentences and the babble are perceived to be separated younger native-English listeners have significantly lower thresholds than the older natives-English listeners, who, in turn, have lower thresholds than the nonnative-English listeners. This suggests that when target and masker are co-located, normal-hearing older, and younger native-English listeners do not differ with respect to speech recognition in a background of babble, but that both groups have lower speech-recognition thresholds than do young nonnative-English listeners. However, speech recognition in the presence of spatial separation is better for younger than for older native-English listeners.

Overall, the results of the R-SPIN word recognition task show that when there are cues to spatially separate the target from the masker, word recognition in older native-English listeners and young nonnnative-English listeners is inferior to that of younger native-English listeners. When listeners of those two groups attempt to communicate in real-life situations where sound sources are often spatially separated, they will experience greater difficulty with respect to word recognition. In older native-English listeners, this increased difficulty most likely is due to the reduction in the quality of the bottom-up information leading to word recognition. In young nonnative-English listeners, difficulties in word recognition are likely due to the increased difficulty they experience in segregating language streams in their L2 (Cooke et al., 2008; Ezzatian et al., 2010).

### Dialog comprehension results

The dialog comprehension results demonstrated that when R-SPIN thresholds were used to adjust for individual differences in the ability to recognize words without supportive context, no effects due to Type of Location (real vs. virtual), or Group (young native-English listeners, older native-English listeners, young nonnative-English listeners) were found. However, the main effect of Separation was significant even though the SNRs were adjusted based on the R-SPIN thresholds. Listeners on average performed significantly better under spatial separation conditions than when sources were co-located. This suggests that spatial separation (real or perceived) facilitates the comprehension and retention of information obtained from the dialogs even when lexical access is presumably equated across the co-located and spatially separated conditions using the R-SPIN adjustment procedure. For example, spatial separation between the talkers may facilitate the association of the incoming information with a specific talker as well as facilitate its retention. However, it is not possible to determine from the present data the precise mechanisms responsible for this spatial separation effect. One possible reason why the R-SPIN adjustment in the spatial separation condition did not eliminate the Separation effect in the dialogs might be that R-SPIN thresholds were determined for a single voice emanating from the right of the listener with the babble occupying a central location. In the separation condition for the dialogs, however, a voice could be on the left or right depending on who was speaking, with the babble emanating from the center. Hence, spatial separation in the dialog condition could have facilitated switching attention back and forth from the right to left depending on who was speaking. The fact that the R-SPIN test did not require the listener to switch attention from one side to other may explain why it was not successful in eliminating the Separation effect in the dialog portion of this study.

The results from the younger and older adults in the real location conditions of the present study were found to be consistent with the results from the equivalent conditions in the Murphy et al. (2006) study. The age difference found when we combined the real location conditions of these two studies supports the hypothesis that older adults might not be as good as younger adult at using the full range of interaural cues to either obtain or maintain stream segregation when sources are separated in space. Older adults frequently need to communicate in multi-talker daily situations taking place in a noisy environment, and naturally they have to do so without any SNR adjustments to accommodate for any individual age-related changes in hearing. The results described here emphasize the notion that in addition to age related difficulties in word recognition, older adults might have a limited toolbox of acoustic cues to assist them when attempting to meet a speech comprehension challenge in real-life situations (e.g., listening to a movie in a surround sound environment). This reduction in the ability to benefit from the acoustic cues provided by physical separation among sound sources is likely to have even greater implications in the presence of a hearing impairment (Neher et al., 2009).

### The relationship between age, linguistic competence, and speech comprehension performance

Given the differences in word recognition across groups and acoustic situations found in the current study, we might expect that different processes are differentially engaged in order to compensate for the specific individual deficits in word recognition when listening to dialog. To examine this we took the two measures of linguistic competence and looked to see the extent to which those measures were correlated with performance under each of the acoustic settings used in the current study. This examination, which was done for both the R-SPIN results as well as for the dialog comprehension results separately, can be used to help identify the relative importance of bottom-up and top-down influences on speech comprehension, and how the pattern of interactions among these factors are modulated by the nature of the acoustic scene.

Specifically, we looked at each of the six groups (12 younger natives in the separation condition; 12 younger native-English listeners in the no-separation condition; 12 young nonnative-English listeners in the separation condition; 12 young nonnative-English listeners in the no-separation condition; 12 older native-English listeners in the separation condition; and 12 older native-English listeners in the no-separation condition) to determine the contribution of the two linguistic measures to performance (number of questions correctly answered) within each group and condition (see Appendix A for further details). This analysis showed that both the vocabulary and the reading comprehension tests results were related to the average number of questions correctly answered (*r* = 0.32, *p* = 0.007, and *r* = 0.38, *p* < 0.001, for reading comprehension skill and vocabulary knowledge, respectively), but that these slopes did not differ across groups. More interestingly, the results of the analysis indicated that there was a significant correlation between vocabulary knowledge and the number of dialog questions correctly answered under the virtual location conditions but not under the real spatial location conditions (see Figure 7), and that this interaction between the Type of Location and Vocabulary was significant (the slopes of the lines differed significantly from each other). We would like to suggest a hypothesis which could explain this finding. An early stage in speech comprehension involves obtaining lexical access to the meaning of words. It has been shown that both bottom-up and top-down processes are involved in word recognition. We can hypothesize that the degree to which top-down processes are engaged in lexical access is modulated by acoustic factors. When listening is relatively easy, we might expect successful lexical access with minimal assistance from top-down processes. However, when listening becomes difficult, we might expect that the top-down processes involved with lexical access to be more fully engaged. When sources are located virtually in space using the precedence effect, they give the impression that their location is diffuse, and there are fewer acoustic cues that can be used to segregate the different acoustic streams than when source location is real (Freyman et al., 1999; Li et al., 2004). Therefore, it is reasonable to expect that under such conditions, obtaining and maintaining stream segregation will be more demanding, and it is possible that the bottom-up processes involved in lexical access will be less reliable because of occasional intrusions from the competing streams. The R-SPIN results indicate that word-recognition is indeed more effortful under virtual as opposed to real location conditions (on average, 50% thresholds are 1.56 dB higher for virtual than for real location conditions), and might require a greater engagement of top-down lexical processes in order to maintain word-recognition accuracy. Hence we would expect that the relative contribution of top-down processes to lexical access to be greater for virtual than for real spatial location conditions. When there is relatively little need to draw on top-down, knowledge-driven processes to obtain lexical access, we would expect a small or negligible contribution of individual differences in the efficacy of these processes to performance. However, when the draw on top-down processes is heavy, then we would expect that some of the variance in performance to be accounted for by individual differences in the efficacy of these processes. Hence we might expect that the contribution of vocabulary knowledge to dialog comprehension performance to increase with level of listening difficulty, as it appears to do in this experiment.

It is also interesting to speculate on the reasons why the relationship between reading comprehension skill and dialog comprehension performance did not differ between real and virtual location conditions (see Appendix A). One possibility is that the linguistic and cognitive skills tapped by the reading comprehension measure are separate from those involved in lexical access. Bottom-up lexical access in these experiments is obtained exclusively through the auditory channel. We can hypothesize that reading comprehension taps higher-order processes that are modality independent and are related to the integration of information, extraction of themes, etc., and therefore are unlikely to be as affected by parameters of the acoustic scene such as whether the location type is real or virtual. On the other hand, we did find group differences with respect to the relationship between reading comprehension and the number of dialog questions answered correctly (Figure 8). Specifically reading comprehension was found to be positively and significantly correlated with performance only in younger native-English listeners

The fact that, in young native-English listeners, individual differences in reading comprehension skills account for a significant portion of the variance in the number of dialog questions correctly answered, indicates that there are higher-order, modality-independent skills that contribute to both reading and listening comprehension in this population. The lack of correlation in older native-English listeners suggests that listening comprehension in difficult listening situations depends on a different set of modality-specific processes that are engaged to compensate for the loss of fidelity in the auditory processing system. In other words in younger native-English listeners, listening comprehension is akin to reading comprehension once lexical access has been achieved. In older native-English listeners and in young nonnative-English listeners, the same degree of listening comprehension (the number of dialog questions correctly answered did not differ across groups) appears to be achieved in a different way. This is consistent with the notion that different neural circuitry supports speech comprehension in different populations (e.g., Harris et al., 2009; Wong et al., 2009).

There may be different reasons why reading comprehension appears to contribute little to individual differences in performance in the older native-English listeners and young nonnative-English listeners. The Nelson-Denny reading comprehension test was developed and standardized for younger native-English listeners and might not be as valid when testing other populations such as older adults or nonnative-English speakers. Because the Nelson-Denny is a time-limited task, it might not adequately reflect individual differences in reading comprehension in older adults whose reading speed is substantially slower than that of younger adults (Rodríguez-Aranda, 2003). It is also unlikely to be a good measure of individual differences in reading competence in young nonnative-English adults either because they too are likely slower readers, or because this test does not adequately gauge the linguistic and cognitive skills used by nonnative-English speakers in comprehending written language. In addition, the draw on attentional resources in young nonnative-English speakers may be higher than in younger and older native-English speakers because lexical access in nonnative speakers most likely requires the activation and integration of information from both their L1 and L2 lexicons (Kroll and Steward, 1994). Moreover, the execution of the higher-order tasks involved in listening comprehension by L2 listeners, such as extracting themes, integrating information with past knowledge, and storing this information for future use may partially be executed in their L1. With respect to older adults, a number of cognitive aging theorists hypothesize that they have a more limited pool of attentional resources than do younger adults (Craik and Byrd, 1982). Alternatively, age-related changes in hearing may place a greater demand on attentional resources in older than in younger adults. Either or both of these factors would result in a greater degree of attentional focus within the auditory domain in older native listeners compared to younger native listeners. As a result, speech comprehension in older adults may depend more on processes that are specific to the auditory modality when listening becomes difficult.

**Figure 9:**
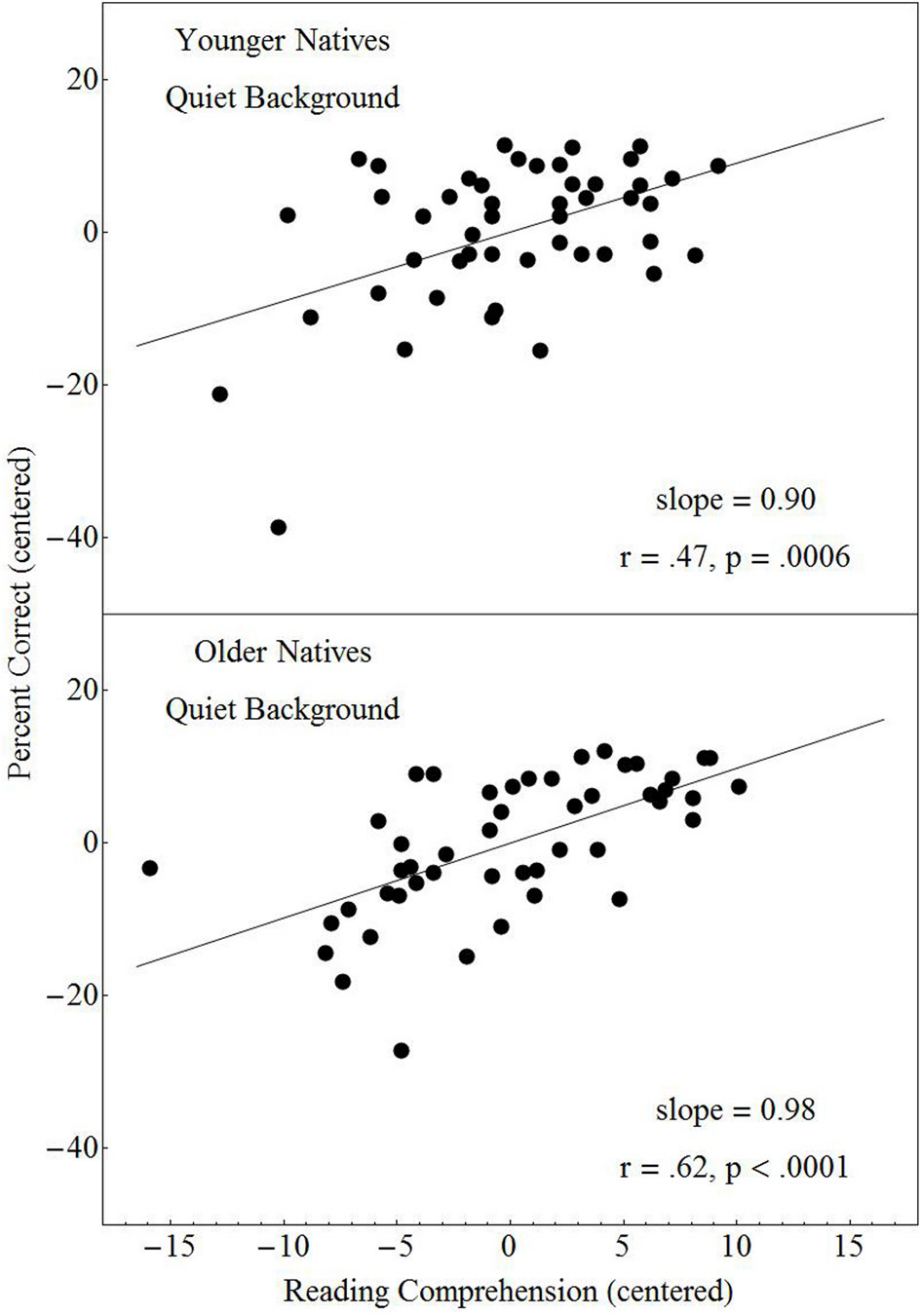
**Percentage of correctly answered dialog questions plotted against the individual performance on the Nelson-Denny reading comprehension test after the contribution of vocabulary to performance had been removed when testing was conducted in quiet in the Murphy et al. (2006) study**. Both the adjusted number of questions answered and the Nelson Denny scores are centered within each group. A least squares regressions line is presented for each of two groups.

To determine whether the failure to find a relationship between reading comprehension and performance in older native-English listeners when listening becomes difficult reflects an increased dependence on modality-specific processes in listening comprehension tasks, we examined the contribution of the reading comprehension performance to the number of correctly answered questions when older native-English listeners were asked to perform a similar task under less demanding perceptual conditions. As previously mentioned, in a study conducted by Murphy et al. (2006), both younger and older listeners were asked to listen to the same two-talker conversations under different acoustic setting, one of which was in quiet. We tested the contribution of vocabulary and reading comprehension to the dialog comprehension performance in quiet (see Appendix B for a detailed description of the analysis conducted) and found that the least squares regressions of the adjusted percentage correct scores against reading comprehension for both the younger and older participants were highly significant (see Figure 9). Hence, under perceptually easy listening conditions, reading comprehension is as strongly related to performance in older native-English listeners as it is in younger native-English listeners. The results of this analysis is consistent with the hypothesis that the lack of a significant contribution of reading comprehension to performance in older adults in noise reflects an increased dependence on modality specific processes when listening becomes difficult.

### Toward a general model of resource allocation in speech comprehension

The differential contribution of vocabulary to dialog performance under virtual vs. real location conditions suggests that difficult listening conditions require that attentional resources be deployed in aid of scene analysis and word recognition. In addition, the relative weight given to bottom-up and top-down processes contributing to lexical access may be shifted in favor of top-down influences when listening becomes difficult. Previous theories such as the Ease of Language Understanding Model (ELU) have proposed that lexical access is impeded or slowed when listening becomes difficult (Rönnberg et al., 2013). Hence, more attentional and working memory resources are required to support lexical access. The current results suggest that the demand on such resources is modulated by the nature of the acoustic scene, which, in turn, affects the engagement of the more central, modality independent cognitive resources involved in language comprehension. Let us assume that the virtual location conditions require additional attentional resources be deployed to locate the diffused sources in space, depleting the pool of the resources available for phoneme identification and bottom-up lexical access. The notion here is that with real spatial location, the task of locating the stimuli is easy whereas virtual localization requires a larger amount of attentional processing. Now consider the problem facing the executive. When full auditory attentional resources can be devoted to lexical access and the bottom-up acoustic information is reliable and sufficient, the executive will trust the output from bottom-up lexical processing, and give less weight to top-down, knowledge-driven factors such as vocabulary knowledge. However, when lower-level attentional resources are required to locate the sound sources, this additional burden reduces the reliability of the information produced through bottom-up lexical processes. In that case, the executive may places more weight on the top-down processes involved in lexical access to compensate for the missing or corrupted bottom-up information.

The hypothesis presented here suggests that depending on the acoustic scene the listening strategy may change the relative engagement of the different processes involved in speech comprehension. The idea that listeners may systematically downplay the contribution of acoustic detail and increase their reliance on lexical-semantic knowledge has been previously suggested by Mattys et al. (2009, 2010) and Mattys and Wiget (2011. Mattys et al. (2009, 2010), Mattys and Wiget (2011) demonstrated a shift which they refer to as a cognitive-load-induced “lexical drift” in cases of high cognitive load (CL) due to an additional secondary task even when no actual energetic masking or additional auditory information is involved (e.g., a secondary visual task). For example, Mattys and Wiget (2011) measured the magnitude of lexical bias on phoneme identification under CL and no CL by adding a secondary visual search task to increase CL. Their results suggested that the CL interferes with detailed phonetic analysis which leaves the listener with impoverished encoding of the auditory input and a greater need to rely on lexical knowledge in order to compensate for the missing information. The collaborative evidence provided by Mattys et al. (2009, 2010) as well as by Mattys and Wiget (2011) and the current study support the existence of a dynamic rather than stationary processing strategy which changes depending on the listening situation, and the age and linguistic status of the listener.

Individual differences in top-down lexical knowledge, as indexed by the Mill-Hill vocabulary test, may be expected to account for a greater proportion of the variance in speech comprehension when the accuracy of the bottom-up processes involved in lexical access is compromised by listening difficulty or by age. Hence we would not expect to find Mill-Hill scores to be related to comprehension in good listening conditions with only a single talker. However, as the listening situation becomes more complex and harder to analyze (competing sound sources, diffused virtual locations rather than compact coherent ones, etc.), the more likely it is that top-down lexical processes will be engaged, and individual differences in speech comprehension to be related to measures of top-down lexical processing. Note also that the complexity of the listening situation need not affect processes subsequent to word recognition. Hence measures indexing the contribution of higher-order processes involved in language comprehension (e.g., integration of information across talkers and with stored knowledge) might not be affected by the acoustic parameters of the auditory scene.

The behavioral evidence that the nature of the acoustic scene, and the age and linguistic competence of the listener, modify the engagement of different auditory and cognitive processes involved in speech comprehension is consistent with recent findings from brain-imaging studies. These studies demonstrate that the degree to which the different neural networks involved in speech comprehension are activated, is modulated by the degree of stimulus complexity, type of task, and age. Previous neuroimaging studies which attempted to map the brain areas involved in speech perception and comprehension demonstrated a frontal-temporal network in which temporal regions subserve bottom-up processes, whereas frontal regions subserve top-down processes (Zekveld et al., 2006). This network seems to be differentially activated depending on the nature of the auditory stimuli and the complexity of the task (Benson et al., 2001; Zekveld et al., 2006). In addition, neural activation seems to not only be affected by the characteristics of the stimuli and task, but also by the characteristics of the listeners as well. Harris et al. (2009) examined the performance of both younger and older adults on word recognition task in which the intelligibility of the stimuli was manipulated using low-pass filtering. Their results showed no age differences in the auditory cortex but differences were found in the anterior cingulate cortex which is presumed to be associated with attention. Age related differences were also found in the Wong et al. (2009) study in which younger and older adults were asked to identified single words in quiet and in two multi-talker babble noise conditions (SNR = 20, −5). The fMRI results for older adults showed reduced activation in the auditory cortex but increased activation in the prefrontal and precuneus regions which are associated with working memory and attention. The increased cortical activities in the general cognitive regions were positively correlated with the behavioral results in the older adults. Wong et al. interpreted this correlation, as well as a more diffused activation involving frontal and ventral brain found in the older adults, as an indication of a possible compensatory strategy used in older age. These studies provide evidence for possible age related changes in the involvement of the different brain regions engaged in speech recognition in noise. As Scott and McGettigan (2013) note in their recent review of the neural processing of masked speech, “Further outstanding challenges will be to identify cortical signatures that are masker specific and that might be recruited for both energetic/modulation masking and informational masking,…and address the ways that aging affects the perception of masked speech while controlling for intelligibility (page 65, last paragraph)”.

In general then, we would expect that the auditory and cognitive processes that are engaged in speech comprehension to be modulated by a number of factors including but not limited to: (1) the complexity of the auditory scene, (2) the nature of the speech material, (3) the task demands placed on the individual, and (4) individual differences in the auditory, linguistic, and cognitive skills and knowledge available to the listener. Future studies which will further examine how one or more of these factors modulate the contribution of auditory and cognitive processes are required. It could be interesting, for example, to conduct a similar study using other background noises such as speech spectrum noise or competing conversations, which differ in the levels of energetic and informational masking created, to further explore the effect masking type may have on the involvement of the different processes which support speech comprehension.

### Conflict of interest statement

The authors declare that the research was conducted in the absence of any commercial or financial relationships that could be construed as a potential conflict of interest.

## Footnotes

^1^These two measures were chosen to tap two different levels of linguistic and cognitive competence. We might expect a vocabulary measure to be related to processes involved with lexical access, whereas reading comprehension would likely tap additional higher-order modality independent processes involved in language comprehension. Other, more specific cognitive and linguistic skills could also be investigated in future studies.

^2^Analyses were done for both the R-SPIN thresholds and the number of correct answers, in which the participant was excluded completely. All the results which were statistically significant in the analyses reported in this paper remained significant and all the results which were statistically insignificant remained as such. We have chosen to replace this one data point with the group average in order to minimize the loss of data.

## Appendix A

### Evaluating the contribution of vocabulary and reading comprehension to R-SPIN threshold and the average percentage of questions correctly answered in the current study

We first checked to see whether vocabulary and/or reading comprehension was related to the average percentage of questions correctly answered by the 72 participants in the experiment. In this and the other tests conducted here we centered the vocabulary and reading comprehension scores within each of the six groups by subtracting the mean score of a group from each of the measures in the group. The six groups were: the 12 younger native-English listeners in the separation condition; 12 younger native-English listeners in the no-separation condition; 12 young nonnative-English listeners in the separation condition; 12 young nonnative-English listeners in the no-separation condition; 12 older native-English listeners in the separation condition; and 12 older native-English listeners in the separation condition. We also centered the average percentage of questions answered correctly in each of these six groups. Centering in this fashion allowed us to evaluate the relative contribution of vocabulary to performance within each group of participants. A regression analysis found a significant correlation between both vocabulary and performance (*r* = 0.38, *p* < 0.001) and reading comprehension and performance (*r* = 0.32, *p* = 0.007).

After finding that both vocabulary and reading comprehension were related to average percentage of questions correctly answered, we then investigated whether the relationship between the two language measures and the dependent variable interacted with one or more of the three factors. Specifically, we tested three hypotheses concerning the slopes of the lines relating these two measures to percent correct. Before conducting this analysis, we centered the percent correct scores within each of the 12 conditions (2-Separations × 3 Groups × 2 Types of Spatial Location) in order to be able to compare the relative contribution of vocabulary and reading comprehension across these 12 conditions. Because both of the measures of language ability and the dependent variable were centered in all conditions and groups, the linear relationship between the individual measures and the dependent variable in a condition was specified by a single parameter, namely the slope of the line relating the measure to performance. Hence, the full model is specified by

(A1) $$Y_{i,j,k,m}=A_{i,\;j,k}V_{i,j,m}+B_{i,j,k}R_{i,j,m}+e_{i,j,k,m}$$

where *Y*_*i,j,k,m*_ is the centered percent correct score in the *i*th level of Separation (voices separate vs. co-located), *j*th Group (younger native-English listeners, older native-English listeners, young nonnative-English listeners), *k*th Type of Location (real vs. virtual), of the *m*th individual in Group *j* and Separation Condition *i*. *V*_*i,j,m*_ and *R*_*i,j,m*_ are the centered vocabulary and reading scores for the *m*th individual from Group *j* and Separation Condition *i*, respectively, and the *e*_*i,j,k,m*_ are assumed to be random normal deviates with mean = 0, and standard deviation = σ.

To test whether the relationships between the vocabulary and reading comprehension measures and the dependent variable were independent of the Type of Location (real vs. virtual), we defined and fit a model in which

(A2) $$Y_{i,j,k,m}=A_{k}V_{i,j,m}+B_{k}R_{i,j,m}+e_{i,j,k,m}.$$

This model allows for the slopes relating the two language measures to the dependent variable to differ only between the real and virtual spatial location conditions. The best-fitting least-squares parameters of this model are the *a*_*k*_ and the *b*_*k*_. We then tested the null hypothesis that *A*_*k* = 1_ = *A*_*k* = 2_ after adjusting the dependent measure for the estimated contribution of reading comprehension, *R*, to performance. In this adjusted model the dependent variable is *Y*′_*R,i,j,k,m*_ = *Y_i,j,k,m_* − *b_k_R_i,j,m_* This null hypothesis was rejected [*F*_(1, 142)_ = 4.70, *p* = 0.03], indicating that the relationship between the centered vocabulary scores and the dependent variable differed between the real and virtual spatial location conditions. However, when the dependent variable was adjusted for the contribution of vocabulary to performance (*Y*′_*V,i,j,k,m*_ = *Y_i,j,k,m_* − *a_k_V_i,j,m_*), the null hypothesis that *B*_*k* = 1_ = *B*_*k* = 2_, could not be rejected [*F*_(1, 142)_ < 1].

To test whether the relationship of both vocabulary and reading comprehension to performance differed between the no-separation and separation conditions, we first averaged over the Within-Subject factor to arrive at a full Between-Subjects model

(A3) $${\bar{Y}}_{i,j,m}=A_{i,j}V_{i,j,m}+B_{i,j}R_{i,j,m}\;+{\bar{e}}_{i,j,m}.$$

We then defined a model in which

(A4) $${\bar{Y}}_{i,j,m}=A_{i}V_{i,j,m}+B_{i}R_{i,j,m}+{\bar{e}}_{i,j,m}.$$

This model allows for the slope relating the language measures to the dependent variable to differ only between the situations in which the two voices were separated vs. when they were co-located. We then tested the null hypotheses that *A*_*i* = 1_ = *A*_*i* = 2_ after correcting for the contribution of reading comprehension to performance in the same fashion as described above. We also tested the null hypothesis that *B*_*i* = 1_ = *B*_*i* = 2_ after correcting for contribution of vocabulary to performance. Neither null hypothesis could be rejected [*F*_(1, 70)_ < 1 for *A*_*i* = 1_ = *A*_*i* = 2_, and *F*_(1, 70)_ = 1.42, *p* = 0.24 for *B*_*i* = 1_ = *B*_*i* = 2_].

To test whether the contribution of vocabulary and reading comprehension to the dependent variable differed among the three groups, we first averaged over the Within-Subject factor and defined a model in which

(A5) $$\;{\bar{Y}}_{i,j,m}=A_{j}V_{i,j,m}+B_{j}R_{i,j,m}+{\bar{e}}_{i,j,m}.$$

This model allows for the slopes relating the two language measures to the dependent variable to differ among the three groups (younger native-English listeners, older native-English listeners, young nonnative-English listeners). We then tested the null hypotheses that *A*_*j* = 1_ = *A*_*j* = 2_ = *A*_*oj* = 3_ after correcting for reading comprehension, and *B*_*j* = 1_ = *B*_*j* = 2_ = *B*_*j* = 3_ after correcting for vocabulary. The null hypothesis that *A*_*j* = 1_ = *A*_*j* = 2_ = *A*_*j* = 3_ could not be rejected [*F*_(2, 69)_ < 1], but the null hypothesis that *B*_*j* = 1_ = *B*_*j* = 2_ = *B*_*j* = 3_ was rejected [*F*_(2, 69)_ = 6.23, *p* < 0.01] Because the relationship between the reading comprehension score and percent correct (adjusted for the contribution of vocabulary) differed across the three groups, we tested three sub-hypotheses: (1) younger native-English listeners' slope = young nonnative-English listeners' slope; (2) younger native-English listeners' slope = older native-English listeners' slope; and (3) young nonnative-English listeners' slope = older native-English listeners' slope. Only the difference between younger native-English listeners' and young nonnative-English listeners' slopes was significant [*F*_(1, 69)_ = 12.37. *p* < 0.01] at the 0.05 level after applying a Bonferroni correction.

It should be pointed out that we obtained exactly the same pattern of results when we independently examined the contributions of reading comprehension and vocabulary to performance, that is, without correcting for the effect of the other language variable on performance.

Recall that R-SPIN thresholds were determined in two conditions: (1) when the precedence effect was used to determine the locations of the voices and babble (virtual location), and (2) when the voices and babble were played over individual loudspeakers (real location). We examined whether either the centered vocabulary measures or the centered reading comprehension scores were related to both sets of centered R-SPIN thresholds. In conducting these tests, we eliminated the young nonnative-English individual in the no-separation group whose R-SPIN threshold for the virtual location condition was more than 3 standard deviations above the mean of that individual's group. None of these four correlations approached significance (*p* > 0.2 in all four cases).

## Appendix B

### Evaluating the contribution of vocabulary and reading comprehension to performance in the quiet conditions of the Murphy et al., 2006 study

The same vocabulary and reading comprehension measures taken on the participants in this study were also taken on the 96 participants (48 young, 48 old) in the Murphy et al. (2006) study, which used the same two-person plays. Four experiments (12 younger and 12 older participants in each experiment) were conducted in both quiet and babble. In this analysis, the dependent variable was the percentage of questions answered in the quiet part of all four experiments. The vocabulary, reading comprehension, and number of questions answered correctly were first centered in each of the four experiments for the two age groups. The full model in the analysis of these data was

(A6) $$Y_{i,j,m}=A_{i,j}V_{i,j,m}+B_{i,j}R_{i,j,m}+e_{i,j,m}$$

where *Y*_*i,j,m*_ is the centered average percentage of questions correctly answered by the *m* participants in the quiet conditions for Age Group *j* of Experiment *i*, and *V, R, A, B* and *e* are defined as above. We then defined and fit a model in which the *A* and *B* coefficients were the same for all four experiments and differed only with respect to the Age Group to which the participants belonged. Specifically, we defined and fit the model

(A7) $$Y_{i,j,m}=A_{j}V_{i,j,m}+B_{j}R_{i,j,m}+e_{i,j,m}$$

We then tested and failed to reject the null hypothesis that *B*_*j* = 1_ = *B*_*j* = 2_ after adjusting for the contribution of vocabulary to performance as described above [*F*_(1, 94)_ < 1]. Least Squares regressions of the adjusted percentage correct scores against reading comprehension for both the younger and older participants were highly significant (slope_young_ = 0.90, *r*_young_ = 0.47, *p* = 0.0006; *slope_old_* = 0.98, *r*_old_ = 0.62, *p* < 0.0001).

After adjusting the centered average percentage of questions correctly answered for the contribution of reading comprehension, there was no evidence of any relationship of the dependent variable to vocabulary (slope_young_ = 0.04, *r*_young_ = 0.01, *p* > 0.5; slope_old_ = 0.72, *r*_old_ = 0.21, *p* > 0.15).
